# Automated quantification of arterial stenosis on CE-MRA by using a deformable vascular tubular model

**DOI:** 10.1186/1532-429X-13-S1-P365

**Published:** 2011-02-02

**Authors:** Avan Suinesiaputra, Patrick JH Koning, Elena Zudilova-Seinstra, Johan HC Reiber, Rob J van der Geest

**Affiliations:** 1 Dept. of Radiology, Leiden University Medical Center, Leiden, Netherlands; 2 Section of Computational Science, University of Amsterdam, Amsterdam, Netherlands

## Introduction

Accurate arterial stenosis quantification is important for the decision of a proper treatment in patients suffering atherosclerotic disease. We have developed an automated arterial stenosis quantification method by using a deformable tubular 3D model that fits into luminal vasculature particularly in severe stenoses.

## Purpose

To provide an automated analysis of arterial stenosis grading with minimal user-interaction.

## Methods

Contrast-enhanced MRA from 21 patients were included. MR images were acquired using a 1.5T MRI scanner with a spoiled 3D FLASH acquisition and a 4x2 circularly polarized phased-array neck coil. Four consecutive 3D images were acquired starting at approximately 3s after the administration of 0.1 mmoL/kg gadolinium. Subtraction images were generated to improve vessel-to-background contrast. To demonstrate the methods’ robustness against image noise, nine subtraction images were included.

Curved multiplanar reformatted image slices were generated perpendicular to the vessel direction and an expert drew luminal contours on these slices. A full-width half-maximum criterion was applied to maintain the contour consistency, particularly for low vessel-to-lumen contrast area.

**Figure 1:**
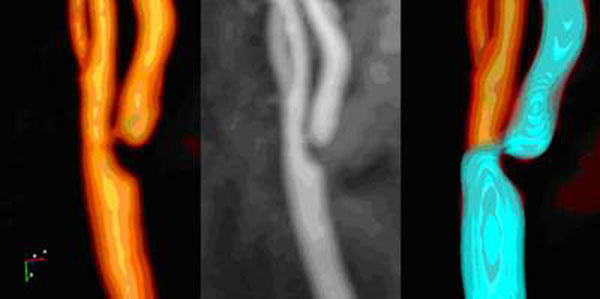
A segmentation result (blue surface). The middle figure is MIP image.

The user defined the artery section of interest by placing proximal and distal points. Subsequently, an automated detection of the vessel pathline was initiated. When the detection was not fully successful, additional intermediate points can be placed or curves can be drawn interactively to create forbidden planes. Subsequently, a tubular model was automatically defined along the pathline and an iterative fitting process was performed to move the control points to fit into the lumen based on the image gradient (Fig. 1 shows a segmentation result). Results of automated image segmentation were compared to the manually traced contours.

## Results

**Figure 2:**
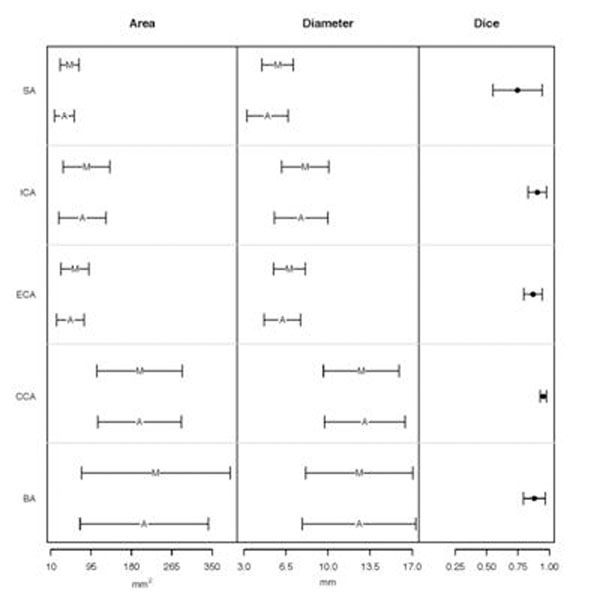
Comparisons between manual (M) and automated (A) in stenosed (SA), internal (ICA), external (ECA), common (CCA) and bifurcation (BA) sections.

**Figure 3:**
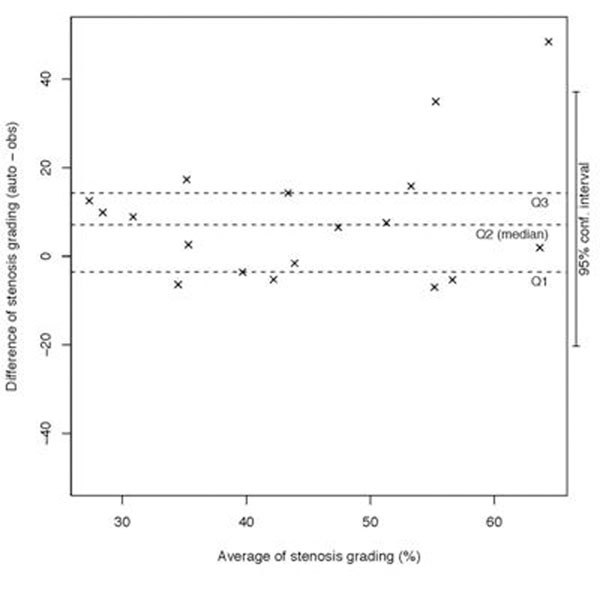
Stenosis grading agreement.

Excellent linear correlations were found on cross-sectional area measurement (r=0.98, p<0.05) and on luminal diameter (r=0.98, p<0.05). Figure 2 shows some comparisons between manual and the automated method. Slight stenosis grading overestimation by the method was observed with a median difference of 7.06% (Fig. 3). Reproducibility was high with a difference between two repeated measurements of only 0.19±7.12%.

## Conclusions

The automated method shows accuracy and reproducibility suitable for clinical application in the analysis of CE-MRA for artery structures. The method requires minimal user-interaction, which saves the processing time of the analysis during the daily routine assessment.

